# Delayed medical consultation and influencing factors in elderly patients with obstructive sleep apnea syndrome

**DOI:** 10.3389/fmed.2025.1555926

**Published:** 2025-07-03

**Authors:** Qihui Sun, Xiaoying Shi

**Affiliations:** ^1^School of Nursing, School of Health, Wuhan University, Wuhan, Hubei, China; ^2^Department of Emergency, Yubei District People’s Hospital of Chongqing, Chongqing, China

**Keywords:** sleep apnea syndromes, health services accessibility, health services for the aged, cross-sectional survey, influencing factors

## Abstract

**Objective:**

This study aims to examine the prevalence of delays in medical consultation and the associated factors among elderly patients with obstructive sleep apnea syndrome (OSA) through a cross-sectional survey design.

**Methods:**

A total of 276 elderly OSA patients were recruited from the sleep monitoring unit of a tertiary hospital in Shanghai, following overnight assessment with the Apnealink portable monitoring device between 2021 and 2022. Data were collected through a general demographic questionnaire, the OSA Knowledge, Attitude, and Practice (KAP) questionnaire, the Perceived Barriers to Medical Consultation Scale, and the Life Satisfaction Scale to evaluate medical consultation delays and their correlates.

**Results:**

Among the 276 elderly OSA patients included in the analysis, the shortest recorded delay in seeking medical attention was 0.20 years, while the longest exceeded 40 years, demonstrating that all patients experienced some degree of delay, with a 100% incidence rate. Factors influencing medical consultation delays were identified in the following order: perceived barriers to medical consultation, levels of KAP, clinical symptoms such as excessive daytime sleepiness and morning xerostomia, life satisfaction, and the method of healthcare payment.

**Conclusion:**

Elderly OSA patients mainly with a secondary or higher education and moderate- or high-income universally face varying degrees of delays in seeking medical care, reflecting an overall concerning trend in healthcare engagement. It is imperative for healthcare professionals to conduct regular health education initiatives aimed at the elderly population to enhance awareness of OSA, foster a proactive approach to seeking medical care, and promote accurate disease comprehension, thereby improving the likelihood of timely consultation.

## 1 Introduction

China is undergoing a rapid demographic shift characterized by accelerated aging and a marked increase in the proportion of elderly individuals (1). The prevalence of obstructive sleep apnea-hypopnea syndrome (OSA) among older adults has been steadily rising in recent years. However, awareness of the disease remains inadequate, often resulting in delayed medical consultations. Early diagnosis and treatment of OSA may not only help slow age-related decline but also delay the progression of aging-associated diseases, thereby significantly improving health outcomes for the elderly population (2). The economic and societal costs arising from untreated OSA, such as impaired quality of life and reduced productivity, far exceed the current expenses associated with its diagnosis and treatment (3–5). Delays in seeking medical care exacerbate the disease’s progression across all severity levels, leading to prolonged symptoms, worsening conditions, and increased challenges in managing chronic comorbidities in elderly patients. This study employed a cross-sectional survey to evaluate older OSA patients’ understanding of the disease, the current state of medical consultation delays, and identify the factors contributing to these delays.

## 1 Materials and methods

### 2.1 Study participants

A purposive sampling method was used to select 276 elderly OSA patients who underwent overnight monitoring with the Apnealink portable screening device (6) at the sleep monitoring laboratory of a tertiary hospital in Shanghai between 2021 and 2022. The inclusion criteria were: (1) an apnea-hypopnea index (AHI) > 5 and a minimum oxygen saturation (SaO_2_) < 90% as determined by overnight sleep monitoring; (2) age > 60 years; (3) willingness to participate in the study; and (4) normal cognitive and communication abilities sufficient for accurate questionnaire completion. Exclusion criteria were: (1) patients diagnosed with central or mixed sleep apnea, or those whose central respiratory events accounted for more than 50% of total respiratory events (7); (2) a history of psychiatric disorders, communication difficulties, or emotional instability that prevented questionnaire completion; and (3) severe insomnia or the use of sedative-hypnotic medications.

### 2.2 Study methods

Patients were guided by trained personnel to complete a series of questionnaires, including a general information questionnaire, the OSA Knowledge, Attitude, and Practice (KAP) Questionnaire, the Chinese-Perceived Barriers to Health Care-Seeking Decision (PBHSD-C) scale, and the Satisfaction with Life Scale (SWLS).

#### 2.2.1 General information questionnaire

A self-designed general information questionnaire was developed based on a thorough literature review and expert consultations with respiratory specialists. The questionnaire covered demographic and clinical information, including age, sex, marital status, education level, sleep monitoring results, medical payment methods, household income, time interval to initial medical consultation, and self-reported symptoms.

#### 2.2.2 OSA KAP questionnaire

The OSA KAP Questionnaire, developed by Peng (8), has been widely utilized to evaluate patients’ knowledge, attitudes, and practices related to sleep apnea. The questionnaire consists of three sections: (1) knowledge of OSA, (2) attitudes toward OSA, and (3) behaviors related to OSA. The total score ranges from 6 to 89, with higher scores reflecting better awareness and practices. The questionnaire demonstrated excellent reliability, with an overall Cronbach’s α of 0.852, and subscale internal consistency coefficients exceeding 0.7.

#### 2.2.3 Chinese-Perceived Barriers to Health Care-Seeking Decision (PBHSD-C) Scale

The PBHSD scale, originally developed by Al-Hassan and Omran (9), has a Cronbach’s α of 0.74. The Chinese version (PBHSD-C), translated and culturally adapted by Li et al. (10), is designed to measure patients’ perceived barriers to seeking medical care. The scale demonstrated strong validity and reliability, with a content validity index ranging from 0.88 to 1, a Cronbach’s α of 0.74, and item-total correlation coefficients exceeding 0.80.

#### 2.2.4 SWLS

The SWLS, developed by Diener (11), is a widely used unidimensional measure of overall life satisfaction. It has been validated across diverse populations and cultures and demonstrates strong psychometric properties. The scale comprises five items rated on a 7-point Likert scale, with higher scores indicating greater life satisfaction. The Cronbach’s α of the SWLS in this study was 0.886.

### 2.3 Statistical analysis

Data were entered into Excel and analyzed using SPSS 25.0. Continuous variables were tested for normality. Mean comparisons across groups were performed using *t*-tests, while categorical variables were analyzed with chi-square (χ^2^) tests and logistic regression. Statistical significance was set at α = 0.05, with *P* < 0.05 considered statistically significant. Descriptive statistics for categorical variables were reported as frequencies (n) and percentages (%), while continuous variables were expressed as mean ± standard deviation. KAP scores, perceived barriers to healthcare-seeking, and life satisfaction were similarly described using means and standard deviations. Univariate analysis: Parametric tests (*t*-tests and one-way ANOVA) were used for continuous variables that met the assumptions of normal distribution and homogeneity of variance. For variables that did not meet these assumptions, non-parametric tests (Wilcoxon signed-rank test and Kruskal-Wallis test) were used to explore the relationships between demographic characteristics, consultation delay times, and KAP levels in elderly OSA patients. Correlation analysis: Pearson correlation analysis was performed for continuous variables that met normality and homogeneity of variance assumptions. For non-normally distributed variables, Spearman correlation analysis was conducted to examine the relationships among KAP scores, perceived barriers to healthcare-seeking, and life satisfaction. Multivariate analysis: Stepwise linear regression analysis was conducted to identify factors influencing delayed medical consultation, including KAP levels, perceived barriers to healthcare-seeking, and life satisfaction.

## 3 Results

### 3.1 Demographic and disease characteristics

An analysis of the demographic and clinical characteristics of 276 elderly patients with OSA was conducted, utilizing frequencies and proportions. The examined variables included age, sex, marital status, education level, medical payment method, monthly per capita household income, disease-related symptoms, and associated comorbidities (Table 1).

**TABLE 1:** General demographic characteristics of elderly OSA patients (*n* = 276).

Item	Count	Percentage (%)
**Age (years)**		
60–69	180	65.2
70–79	82	29.7
≥80	14	5.1
**Gender**		
Male	156	56.5
Female	120	43.5
**Marital status**		
Unmarried	0	0
Married	248	89.9
Others	28	10.1
**Education level**		
Below primary school	44	15.9
Secondary school	146	52.9
College and bachelor’s degree	86	31.2
Master’s degree or above	0	0
**Payment method for medical expenses**		
Out of pocket	26	9.4
Urban-rural medical insurance	188	68.1
Employee medical insurance	46	16.7
Others	16	5.8
**Average monthly income per family member**		
<2,000	30	10.9
2,000–5,000	188	68.1
>5,000	58	21
**Severity of obstructive sleep apnea**		
Mild	72	26.1
Moderate	94	34.1
Severe	110	39.1
**Self-reported or family–reported symptoms of snoring or apneas**		
No	30	10.9
Yes	246	89.1
**Habit of mouth breathing at night**		
No	128	46.4
Yes	148	53.6
**Symptoms of dry mouth upon waking**		
No	64	23.2
Yes	212	76.8
**Daytime sleepiness and fatigue symptoms**		
No	28	10.1
Yes	162	58.7
Moderate	86	31.2
**Presence of rhinitis, pharyngitis, nasal polyps, or tonsillar hypertrophy**		
No	122	44.2
Yes	104	37.7
Unknown	50	18.1
**Presence of underlying diseases (hypertension, diabetes, heart disease, hyperlipidemia)**		
No	61	22.1
One kind	95	34.4
Two kinds	72	26.1
Three kinds or more	38	13.8
Unknown	10	3.6
**Lifestyle habits of smoking or alcohol consumption**		
No	214	77.5
Yes	62	22.5
**Presence of sleep disorders (insomnia, difficulty falling asleep, frequent awakenings)**		
No	170	61.6
Yes	106	38.4

### 3.2 Status of medical consultation delays among elderly OSA patients

The distribution of delays in seeking medical consultation among the 276 elderly OSA patients was found to be non-normal (*p* < 0.05). The minimum delay recorded was 0.20 years, while the maximum reached 40 years, with a median delay time of 10.07 years (P25 = 5 years, P75 = 18.58 years). Stratification of the delay times into six intervals (0–0.25 years, 0.26–1 year, 1.01–5 years, 5.01–10 years, 10.01–20 years, and 20.01–40 years) indicated that the largest proportion of patients (31.2%) experienced delays ranging from 10.01 to 20 years (Table 2).

**TABLE 2:** Segmentation of medical consultation delays in elderly OSA patients (*n* = 276).

Consultation time (years)	Frequency	Percentage (%)
0–0.25	4	1.4
0.26–1	19	6.9
1.01–5	48	17.4
5.01–10	67	24.3
10.01–20	86	31.2
20.01–40	52	18.8

### 3.3 Univariate analysis of factors influencing medical consultation delays among elderly OSA patients

The univariate analysis revealed that significant daytime sleepiness and fatigue symptoms were statistically significant contributors to the duration of medical consultation delays (χ^2^ = 2.554, *P* = 0.036) (Table 3).

**TABLE 3:** Comparison of medical consultation delays among elderly OSA patients with different demographic and disease characteristics (*n* = 276).

Item	Category	Count (n)	Percentage (%)	Consultation delay time (mean ± SD)	Z/x^2^	*p*
Age	60–69	180	65.2	12.73 ± 9.34	1.775②	0.503
	70–79	82	29.7	12.43 ± 10.87		
	≥ 80	14	5.1	14.81 ± 10.22		
Gender	Male	156	56.5	13.20 ± 10.42	−0.445①	0.656
	Female	120	43.5	12.15 ± 9.02		
Marital status	Unmarried	0	0	0	0.159②	0.453
	Married	248	89.9	12.4 ± 9.28		
	Others	28	10.1	15.81 ± 13.64		
Education level	Below primary school	44	15.9	12.85 ± 11.72	4.837②	0.274
	Secondary school	146	52.9	13.67 ± 10.15		
	College and bachelor’s degree	86	31.2	11.12 ± 7.98		
	Master’s degree or above	0	0	0		
Payment method for medical expenses	Out of pocket	26	9.4	11.95 ± 12.61	4.326②	0.377
	Urban-rural medical insurance	188	68.1	12.98 ± 9.84		
	Employee medical insurance	46	16.7	11.80 ± 9.22		
	Others	16	5.8	14.00 ± 6.35		
Average monthly income per family member	< 2,000	30	10.9	13.92 ± 11.32	5.713?	0.403
	2,000–5,000	188	68.1	13.09 ± 9.91		
	> 5,000	58	21	11.01 ± 8.64		
Severity of obstructive sleep apnea	Mild	72	26.1	14.00 ± 10.31	7.039②	0.18
	Moderate	94	34.1	11.69 ± 10.24		
	Severe	110	39.9	12.83 ± 9.13		
Self-reported or family-reported symptoms of snoring or apneas	No	30	10.9	10.63 ± 10.16	−1.646②	0.1
	Yes	246	89.1	13.00 ± 9.78		
Habit of mouth breathing at night	No	128	46.4	12.63 ± 10.78	−0.871①	0.384
	Yes	148	53.6	12.84 ± 8.97		
Symptoms of dry mouth upon waking	No	64	23.2	13.78 ± 10.67	−0.790①	0.43
	Yes	212	76.8	12.43 ± 9.57		
Daytime sleepiness and fatigue symptoms	No	28	10.1	8.27 ± 7.28	2.554①	0.036*
	Yes	163	58.7	13.45 ± 10.63		
	Moderate	86	31.2	12.88 ± 8.62		
Presence of rhinitis, pharyngitis, nasal polyps, or tonsillar hypertrophy	No	122	44.2	12.38 ± 9.80	0.071②	0.619
	Yes	104	37.7	12.22 ± 8.77		
	Unknown	50	18.1	14.70 ± 11.80		
Presence of underlying diseases (hypertension, diabetes, heart disease, hyperlipidemia)	No	61	22.1	12.47 ± 10.23	0.393②	0.814
	One kind	95	34.4	12.6 ± 8.96		
	Two kinds	72	26.1	13.36 ± 9.56		
	Three kinds or more	38	13.8	14.02 ± 8.59		
	Unknown	10	3.6	11.54 ± 11.35		
Lifestyle habits of smoking or alcohol consumption	No	214	77.7	12.96 ± 9.80	−0.811①	0.481
	Yes	62	22.5	11.99 ± 10.00		
Presence of sleep disorders (insomnia, difficulty falling asleep, frequent awakenings)	No	170	61.6	13.06 ± 9.91	−0.719①	0.472
	Yes	106	38.4	12.24 ± 9.74		

### 3.4 Correlation analysis of medical consultation delays among elderly OSA patients

Correlation analysis demonstrated a significant association between delay time and various health behaviors, as well as perceived barriers to seeking medical care (*p* < 0.05). Furthermore, a strong correlation was observed between delay time, disease knowledge, and life satisfaction (*p* < 0.01) (Table 4).

**TABLE 4:** Correlation between delay in medical consultation and knowledge, attitude, health behavior, perceived barriers, and life satisfaction.

	Delay in medical consultation	Disease knowledge	Disease attitude	Health behavior	Knowledge-attitude-behavior	Perceived barriers	Life satisfaction
Delay in medical consultation	1						
Disease knowledge	−0.145**	1					
Disease attitude	−0.003	0.450**	1				
Health behavior	0.092*	0.054	0.159**	1			
Knowledge-Attitude-Behavior	−0.063	0.866**	0.674**	0.412**	1		
Perceived barriers	0.91*	−0.098**	−0.101**	−0.220**	−0.174**	1	
Life satisfaction	−0.103**	0.407**	0.4423**	0.307**	0.529**	−0.327**	1

**p < 0.01, *p < 0.05.

### 3.5 Multivariate analysis of factors influencing medical consultation delays among elderly OSA patients

A stepwise linear regression analysis was conducted to assess the factors influencing medical consultation delays. The independent variables included demographic characteristics, disease-related data, levels of KAP, along with perceived barriers to care-seeking and life satisfaction. The dependent variable was defined as the delay time in seeking medical consultation among elderly OSA patients.

The regression model identified several significant predictors of delay time: perceived barriers to care-seeking, disease knowledge, daytime sleepiness and fatigue, disease attitudes, life satisfaction, morning dry mouth, rhinitis, pharyngitis, nasal polyps, tonsillar hypertrophy, and medical payment method. It is worth noting that the severity of OSA is not an independent influencing factor for the delay in seeking medical treatment. These variables were integrated into the regression equation, which yielded statistical significance (*F* = 10.796, *p* < 0.0001) (Tables 5, 6).

**TABLE 5:** Stepwise linear regression analysis of factors affecting delay in medical consultation for elderly OSA patients.

Variable name	Assignment method
Age	60–69 = 1; 70–79 = 2; ≥ 80 = 3
Gender	Male = 1; female = 2
Marital status	Unmarried = 1; married = 2; other = 3
Education level	Below primary school = 1; secondary = 2; college and bachelor’s = 3; master’s and above = 4
Severity of disease	Mild = 1; moderate = 2; severe = 3
Payment method for medical expenses	Out of pocket = 1; urban-rural insurance = 2; employee insurance = 3; other = 4
Average monthly income per family member	<2,000 = 1; 2,000–5,000 = 2; > 5,000 = 3
Mouth breathing at night	No = 1; yes = 2
Snoring at night	No = 1; yes = 2
Dry mouth upon waking	No = 1; yes = 2
Daytime sleepiness and fatigue	No = 1; yes = 2; moderate = 3
Presence of rhinitis, pharyngitis, nasal polyps, or tonsillar hypertrophy	No = 1; yes = 2; unknown = 3
Presence of underlying diseases (hypertension, diabetes, heart disease, hyperlipidemia)	No = 1, One kind = 2, two kind = 3, three or more = 4, Unknown = 5
Lifestyle habits of smoking or alcohol consumption	No = 1; yes = 2
Sleep disorders (insomnia, difficulty falling asleep, frequent awakenings)	No = 1; yes = 2
Knowledge-attitude-behavior level	Actual measurement value
Perceived barriers	Actual measurement value
Life satisfaction	Actual measurement value

**TABLE 6:** Regression analysis of factors influencing delay in medical consultation for elderly OSA patients (*n* = 276).

Item	Unstandardized coefficients (B)	Standardized coefficients (β)	*t*	*P*
Constant	0.153	4.357	–	0.035
Perceived barriers	0.31	0.061	0.198	<0.001
Disease knowledge	–0.285	0.057	–0.2	<0.001
Daytime sleepiness and fatigue	2.238	0.547	0.141	<0.001
Disease attitude	0.381	0.106	0.148	<0.001
Life satisfaction	–0.226	0.097	–0.097	<0.05
Dry mouth upon waking	−2.55	0.835	–0.109	<0.05
Presence of rhinitis, pharyngitis, nasal polyps, or tonsillar hypertrophy	1.414	0.498	0.107	<0.05
Payment method for medical expenses	1.21	0.576	0.077	<0.05
Severity of obstructive sleep apnea	1.25	2.31	0.621	0.791

## 4 Discussion

The findings from this study reveal that among the 276 elderly patients diagnosed with OSA, the shortest delay in seeking medical care was 0.20 years, while the longest was over 40 years. This indicates a pervasive issue of consultation delays, with an incidence rate of 100%. These results are consistent with the research conducted by Shang et al. (12). Additionally, the incidence of OSA among postmenopausal women showed a significant increase, reaching levels comparable to those of men in the same age cohort (13), which aligns with the findings of Bonsignore et al. (14) and Thompson et al. (15). A substantial majority of the study population were married (89.9%), corroborating Su’s investigation into the marital status of the elderly within the context of aging (16). This suggests that married OSA patients are more likely to seek medical attention due to the impact on their partners’ sleep quality (17, 18).

Concerning educational attainment, a significant proportion of participants had completed secondary education (52.9%) or higher education (31.2%). This suggests that higher levels of education may correlate with greater awareness of health issues and a more proactive stance toward treatment. This finding is supported by research conducted by Hlaing and Sawunyavisuth, indicating that elderly OSA patients with higher educational backgrounds tend to possess a better understanding of the disease and demonstrate a greater willingness to pursue diagnosis and treatment (19, 20). Furthermore, the study was conducted in an eastern region with rich educational resources, which may have influenced the patients’ educational backgrounds.

The data revealed that 68.1% of elderly OSA patients utilized urban-rural medical insurance, while only 9.4% were self-paying. This suggests that the availability of medical insurance likely encourages more proactive healthcare-seeking behaviors among patients. Medical insurance plays a critical role in lowering barriers to care and alleviating concerns regarding medical expenses. Most participants reported a monthly per capita household income between 300 and 700 dollars (68.1%), with only 10.9% having an income below 300 dollars. This indicates that the majority of patients come from middle-income households, with a relatively low representation of patients from low-income families.

The study found a direct correlation between the perception of barriers to seeking care and the duration of consultation delays. Patients with higher perceived barriers exhibited longer delays in seeking medical attention, echoing findings from Lin (21) and Jiang et al. (22). The item with the highest score regarding perceived barriers was “I prefer to consult my family before visiting the hospital,” underscoring the significant role that family plays in decision-making for this demographic. Elderly patients often refrain from making medical decisions independently, instead opting to seek assistance and advice from family members, a viewpoint supported by Li (23). Research indicates that cultural emphasis on family and collective decision-making discourages individuals from making solitary decisions, often resulting in delays in addressing critical health issues (24). While this collaborative decision-making process may provide psychological comfort and support, it can also lead to delays in urgent decision-making during emergencies.

The advent of digital healthcare services has expanded the information and choices available to patients; however, it may also present new challenges. For elderly patients, the integration of new technologies can introduce operational difficulties and confusion regarding the processing and interpretation of digital information. This “technology anxiety” may compel them to rely more on family members rather than seeking medical help directly (25, 26).

The overall KAP score for elderly OSA patients in this study was (40.27 ± 10.41), which is lower than the findings of Peng (8) (45.66 ± 15.27). While elderly patients demonstrated positive attitudes and higher scores in health behaviors, their knowledge regarding the disease was notably inadequate. This suggests that although they may recognize the importance of OSA treatment, their understanding of the disease’s specifics and potential risks is insufficient, leading to hesitance and delays in seeking professional help. The highest scoring item was “The occurrence of OSA increases with age,” which likely reflects their awareness of worsening symptoms as they age (27). Conversely, the lowest scores were associated with knowledge of OSA’s potential to cause cognitive impairment, treatment-resistant hypertension, and its close association with cerebrovascular diseases. This lack of understanding may hinder patients’ ability to attribute their symptoms correctly and further delay their healthcare-seeking behavior, consistent with findings from Shang et al. (12) and Zhao et al. (28). Enhancing the KAP levels of elderly patients is crucial not only for disease prevention and management but also for fostering timely medical interventions, which can improve early diagnosis and treatment, thereby enhancing disease prognosis (29, 30).

The study indicated that a significant proportion (73.2%) of elderly OSA patients were classified as having moderate to severe disease severity, corroborating findings from Hongyo et al. (27). Furthermore, a negative correlation between excessive daytime sleepiness and consultation delays may be attributed to the more pronounced fatigue and somnolence experienced by elderly patients due to declining physiological functions. Research has indicated that snoring and excessive daytime sleepiness are the most readily perceived symptoms of OSA by patients (31, 32). However, many elderly patients, particularly those on psychotropic medications (13, 33), may not connect their daytime fatigue and drowsiness to OSA, resulting in delays in seeking care. Moreover, studies indicate that excessive sleepiness is less common among patients with mild obstruction (AHI < 15), which may contribute to delays in diagnosis and treatment within this demographic (34).

In the univariate analysis of healthcare-seeking behavior among elderly OSA patients, the variables representing “morning dry mouth symptoms” and “nasopharyngeal diseases” did not initially demonstrate significant effects on consultation delays. However, subsequent multivariate regression analysis revealed these factors as significant after controlling for other potential influences. The negative correlation between “morning dry mouth symptoms” and consultation delays suggests that elderly OSA patients experiencing such symptoms tend to seek medical attention earlier. This may be due to the direct impact of morning dry mouth on their daily comfort and quality of life, making it more noticeable and prompting them to perceive it as a medical concern. Conversely, the positive correlation between “nasal inflammation, pharyngitis, nasal polyps, and tonsillar hypertrophy” and consultation delays may reflect the chronic and mild nature of these conditions, which may not initially command patients’ urgent attention. Consequently, patients may postpone seeking care until symptoms exacerbate or lead to other complications. Furthermore, studies indicate that elderly patients often have poorer awareness of sleep disorders and related symptoms compared to younger individuals (35), which may contribute to delays in seeking care.

Elderly OSA patients reporting higher life satisfaction were more likely to actively seek medical help. This finding aligns with research by Silva et al. (36), which noted that elderly individuals, particularly older women, exhibit a stronger willingness to seek care due to elevated health awareness and life satisfaction. The study reinforces previous literature that highlights a high proportion of moderate to severe OSA patients, who are significantly affected by increased nocturnal arousal indices and pronounced daytime sleepiness (37). Consequently, these individuals are more motivated to seek treatment to alleviate their symptoms. Additionally, studies have reported that a history of nocturia in elderly men serves as a significant indicator of potentially severe OSA, as it negatively impacts sleep quality and overall life satisfaction, thus promoting more proactive healthcare-seeking behaviors (38, 39). This suggests that a history of nocturia in elderly male OSA patients could serve as a robust predictive marker for assessing disease severity and its implications. It has also been noted that OSA-related acute and chronic conditions can adversely affect sleep quality and life satisfaction among elderly patients (40–42), underscoring the critical importance of timely medical intervention, particularly within the elderly population.

In this study, the category labeled as “other” medical payment methods encompasses those not covered by local insurance plans. Patients falling into this category often pursue medical care in different regions, where they may face additional obstacles, including an uneven distribution of healthcare resources. This disparity can lead to prolonged delays in seeking consultations. Such challenges may stem from a lack of readily available resources for the diagnosis and treatment of OSA in their localities, which forces patients to seek care elsewhere, resulting in longer wait times and more complicated coordination efforts. Research indicates that the prevalence of OSA is particularly high among elderly individuals from lower socioeconomic backgrounds (43). The significant costs associated with diagnosing and treating OSA can deter these patients from pursuing necessary care, particularly when financial constraints are a factor. This sensitivity to costs plays a crucial role in shaping the healthcare-seeking behaviors of elderly patients with OSA, especially in scenarios where insurance reimbursement is not an option (5, 18, 44).

This study has several limitations. First, all participants were recruited from a tertiary hospital in Shanghai, which may introduce geographical selection bias and limit the generalizability of the findings. Second, as a cross-sectional study, it cannot establish causality and can only reveal correlations between variables. Additionally, the questionnaire used in this study is subjective and may be influenced by participants’ recall bias or social desirability, affecting the accuracy of the data. Future studies should expand the sample to include more regions, especially rural and western areas, to enhance the representativeness of the results. Longitudinal study designs could be adopted to clarify causal relationships, and a combination of interviews and other data collection methods could be used to reduce the limitations of subjective questionnaires. In addition, considering the small sample size, in order to avoid over—interpretation, subgroup analysis of disease severity was not conducted, which is also one of the limitations of this study.

The findings of this study carry significant practical implications for clinical practice and public health policy. Firstly, the study reveals that delayed medical consultation is widespread among elderly OSA patients, with a wide range of delay durations. This indicates the need for enhanced early screening and diagnosis of OSA, particularly in community health centers, where regular check-ups and health education can improve early disease detection (45). Secondly, the study identifies multiple factors contributing to consultation delays, such as limited disease awareness, financial burden, and psychological barriers. In response to these factors, the following measures are recommended: conducting targeted health education to raise patients’ awareness of OSA and its risks, optimizing medical service processes to reduce obstacles in seeking care, and providing financial assistance information to improve patients’ compliance with medical treatment (46, 47). Additionally, the study highlights the importance of family and social support. Encouraging family members to participate in patients’ health management and offering emotional and practical support, such as assisting with appointments and accompanying patients to medical visits, is advised (48). For policymakers, the results suggest a need to focus on the management and prevention of chronic diseases like OSA. Increasing investment in community health services and improving the diagnostic and treatment capabilities of primary care institutions, such as by equipping necessary monitoring devices and training professional medical staff, is recommended (49, 50). Furthermore, establishing a multi—departmental collaborative intervention mechanism to integrate medical, community, and family resources can collectively improve the timeliness of medical consultation and disease prognosis for elderly OSA patients.

## 5 Conclusion

This study highlights that elderly patients with OSA mainly with a secondary or higher education and moderate- or high-income consistently face varying degrees of delays in seeking medical care, indicating a troubling trend in healthcare engagement. Several factors contribute to these delays, including perceived barriers to care, levels of KAP, symptoms such as daytime sleepiness and morning dry mouth, life satisfaction, and the nature of medical payment methods.

## Data Availability

The original contributions presented in this study are included in this article/supplementary material, further inquiries can be directed to the corresponding author.
